# Catalan ethnoflora: a meta-analytic approach to life forms and geographic territories

**DOI:** 10.1186/s13002-020-00424-x

**Published:** 2020-11-25

**Authors:** Airy Gras, Montse Parada, Joan Vallès, Teresa Garnatje

**Affiliations:** 1 Institut Botànic de Barcelona (IBB, CSIC-Ajuntament de Barcelona), Passeig del Migdia s.n., Parc de Montjuïc, 08038 Barcelona, Catalonia Spain; 2 Laboratori de Botànica (UB)—Unitat associada al CSIC, Facultat de Farmàcia i Ciències de l’Alimentació—Institut de Recerca de la Biodiversitat (IRBio), Universitat de Barcelona, Av. Joan XXIII 27-31, 08028 Barcelona, Catalonia Spain; 3 Secció de Ciències Biològiques, Institut d’Estudis Catalans, Carrer del Carme 47, 08001 Barcelona, Catalonia Spain

**Keywords:** Catalonia, Ethnobotany, Geographic areas, Life forms, Physiographic zones, Traditional knowledge

## Abstract

**Background:**

Catalonia (in the north east of the Iberian Peninsula) is among the most prospected territories in Europe, from the ethnobotanical point of view. The aim of the present paper is to undertake a global analysis in the area considered, including plants, plant life forms, and ethnobotanical data within a physiographic and geographic framework.

**Methods:**

Data from 21 ethnobotanical prospection areas in Catalonia were collected, analyzed, and compared, with the focus on plant life forms and geographic divisions.

**Results:**

A total of 824 taxa constitute the Catalan ethnoflora, and 316 of them are shared by the six physiographic zones recognized in Catalonia. When three major geographic areas are considered (Pyrenean, inland, and littoral), 394 taxa have been reported in only one out of the three areas.

Concerning life forms, phanerophytes and chamaephytes together, i.e., those taxa present all through the year, are the most cited (37.12%).

**Conclusions:**

This first study constitutes a new approach to ethnobotanical data analysis. The results show the particular importance of plants with a large distribution area and plants with available biomass throughout the year. Apart from this, other kind of plants, e.g., those present in only one territory, are of interest for its originality and sometimes for the local significance.

## Introduction

Folk plant use for extremely diverse purposes is inherent to humanity, as suggested by the evidence of their varied employment in ancient times [1] and by the convergence of their uses in both close and distant societies [2, 3]. Ethnobotany [4] is situated at the interface between the social and natural sciences [5] and projects ancestral traditional knowledge regarding biodiversity into the present and future wellbeing of human societies [6]. The majority of ethnobotanical work has been devoted to ethnofloristic prospection [7], with an emphasis on pharmaceutical ethnobotany or ethnopharmacology, probably due to the possibilities of using some of the collected information in the drug development process [8, 9]. Apart from the ethnofloristic approach, comparative and quantitative studies are also relevant in ethnobotany. In the last 30 years, ethnobotanical prospection has been prolific in Catalonia (the north east of the Iberian Peninsula) [10], and today, with 21 well-studied territories, a general perspective on Catalan ethnoflora is possible.

**Fig. 1:**
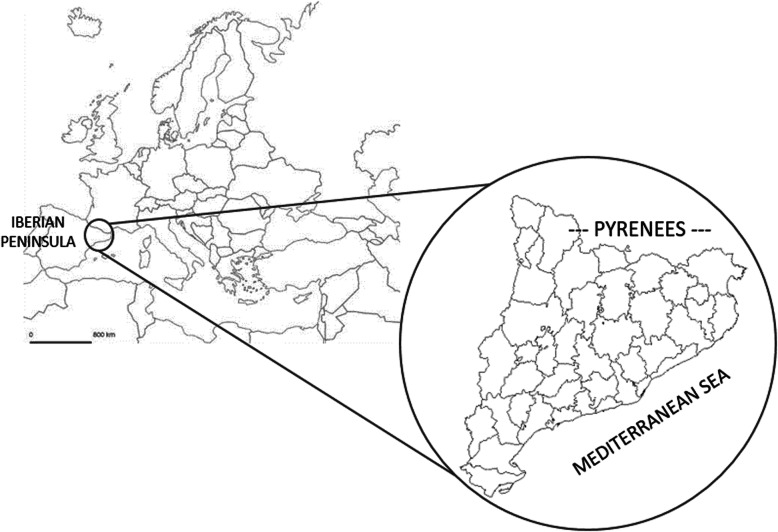
Map of the study area, Catalonia, showing the location within Europe

Catalonia is located in the north east of the Iberian Peninsula (Fig. 1) and occupies an area of 31,895 km^2^. It includes very diverse territories from both the geographic and climatic points of view and is consequently covered by different vegetation types [11, 12]. Catalonia also has a very varied relief, which, from sea level to an altitude of ca. 3150 m, hosts a panoply of landscape types. The Pyrenees and littoral plains occupy a very important portion of the territory, and the rest basically belong to the inner plains, the westernmost being of an arid climate [13]. In this region of high physical geographic diversity, which is associated with a wide range of climatic zones, the plant landscape comprises elements included in the Mediterranean, Euro-Siberian, and Boreo-Alpine regions. The Catalonian landscape contains associations ranging from perennial-leaf *Quercus* communities on the littoral plains to alpine meadows in high mountains, through to forest communities dominated by deciduous-leaf *Quercus*, *Fagus*, *Pinus,* or *Abies*, as well as maquis shrublands, dry plain meadows, and other kind of communities [14].

Catalonia has a population of 7,675,217 inhabitants, unequally distributed. The majority of this population is concentrated around Barcelona and its metropolitan area and other highly industrialized areas [15]. Specifically, 64.19% of the total surface area of the territory holds only 4.44% of the population, with this being 1.12%, less than 16 years ago. In these areas, 62.59% of the municipalities have less than 2000 inhabitants [15]. In some areas, agricultural activity has been replaced by tourism as the main economic activity.

The exodus from rural to urban areas, which took place mainly during the last century, played a key role in the erosion of popular knowledge and also the relationship between people and their natural environment. Exchanges of knowledge between geographically distant areas, such as those produced by transhumance or the *trementinaires*, have now been almost completely lost, but comparisons between areas show the results of this dispersion of knowledge.

In the framework of an essay categorizing the Catalan linguistic area from the point of view of physical geography, Bolòs [16, 17] described several physiographic zones, six of them in Catalonia. These physiographic territories are the basis of the distributional explanations for taxa in the flora of the Catalan countries [14], encompassing the area considered in the present study. (1) Pyrenees: The Pyrenean range is located in the northern part of the studied territory. Three units are usually contemplated due to its wide area and great diversity: the western Pyrenees, located in their entirety outside the Catalan territory; the central Pyrenees; and the eastern Pyrenees. The highest Pyrenean altitude in Catalonia is 3143 m a.s.l. The climate is proper of mountains, including high-mountain areas, with abundant snow precipitation in winter. (2) Ruscinic territory: A maritime plain at the foot of the eastern slopes of the Pyrenees. This territory includes the Rosselló plain in the north and the Empordà plain in the southern part. The zone is generally maritime Mediterranean in character, but the north wind is strong, the winters are somewhat severe and the climate is less moist than the zone immediately to the west and south. (3) Olositanic territory: To the south-west of the Ruscinic zone, linking the Pyrenees to the Catalan coastal range, lies a system of low and moderate-high mountains and small valleys. The fact that rainfall is relatively high and the summer drought not very intense gives rise to transitions between the moist Mediterranean landscape that predominates over a large part of the territory and landscapes which are somewhat Atlantic in character. (4) Catalanidic territory: The outstanding feature of this territory is the great system of the Catalanidic mountains. This complex system alternating low and moderate-high mountains and low depressions, arranged in rows parallel to the Mediterranean coast, extends and stretches over a distance of more than 300 km. The climate is generally maritime, with environmental ranges from temperate and humid conditions to hot and dry. (5) Auso-Segarric territory: Area of gentle relief with ranging from 200 to 1000 m. The climate is largely continental, often with marked temperature inversions the bottom of troughs. (6) Sicoric territory: A low-lying plain, located in the western part of Catalonia. The climate is continental Mediterranean with an arid tendency [16, 17]. Despite this high number of territories for a relatively small surface area, three major areas can be distinguished: Pyrenean and pre-Pyrenean (hereafter abridged Pyrenean), inland, and littoral and pre-littoral (hereafter abridged littoral), defined by geographic and major area criterions.

Raunkjaer described the plant life forms in 1905 for the first time, classifying them based on the position of the plant buds during the seasons with adverse conditions [18]. Following the plant life forms described, phanerophytes are the plants that grow taller than 25–50 cm, or whose shoots do not die back periodically to that height limit; chamaephytes are the plants whose mature branch or shoot system remains perennially within 25–50 cm above ground surface, or plants that grow taller than 25–50 cm, but whose shoots die back periodically to that height limit; hemicryptophytes are the perennial herbaceous plants with periodic shoot reduction to a remnant shoot system that lies relatively flat on the ground surface; geophytes are the perennial herbaceous plants with periodic reduction of the complete shoot system to storage organs that are imbedded in the soil; therophytes are the annual plants, whose shoot and root system dies after seed production and which complete their whole life cycle within 1 year; hydrophytes are the plants that rest submerged under water; and epiphytes are the plants that do not grow in soil but on phanerophytes [18]. This system provides valuable information about the plant availability throughout the year in each territory, and finally, the probability that the plants can easily be found and used by the informants. Other classification systems have been carried out by different authors according to several plants’ characteristics [19, 20], but the Raunkjiaer's system is the most complete and robust one, in the sense it is only one that shows the availability of plants throughout the year. Several ethnobotanical studies include some information about the life form of the plant taxa, but usually they are only devoted to distinguish between annual and perennial or among shrubs, trees, and herbs [21]. These classification systems are less accurate than Raunkjiaer’s, which was also used by other authors [22].

The aim of the present paper is to conduct an extensive meta-analytic study within the area considered in a new approach in interpreting ethnobotanical data. The study includes plant taxa, plant life forms in order to evaluate the influence of the plant availability, and ethnobotanical data in physiographic and geographic frameworks in order to study the possible relationships between geographic factors and traditionally used flora. Given the inexistence, or large scarcity, of studies with this focus, our proposal is to encourage such investigation in other cultural and geographical areas.

## Material and methods

### Databasing

All research information collected in the fieldwork was introduced in our research team’s database, encompassing all the Catalan-speaking territories (www.etnobotanica.cat). During the fieldwork, the method used was the semi-structured interview [23], always taking into account the ethical principles of the International Society of Ethnobiology [24] and with the oral informed consent of the informants [25]. The database includes data directly collected by our team and information from other studies on the concerned territories. The database will be progressively offered in open-access form from the end of 2020, but all data used for this work are available in the Supplementary material file. This database contains all kinds of ethnobotanical data (medicinal, food, and other uses, as well as vernacular names, toxicity, and ecological observations) and allows one to perform various calculations and to make comparisons. Nevertheless, it must be taken into account that ethnofloristic data are dynamic, so that new prospections could affect the results presented herein.

### Data analysis and biases

A total of 824 taxa at specific and infraspecific levels constitute the dataset of the Catalan ethnoflora analyzed, coming from works performed between 1991 and present. We use the term “ethnoflora,” as in other ethnobotanical papers, in the sense of Merriam-Webster dictionary: “the part of the flora of a region used by its human aborigines” (https://www.merriam-webster.com/dictionary/ethnoflora). As for the flora, we consider wild vascular plants, so that, in the current analysis, 17 non-vascular plants, 64 taxa only identified at the generic level, 159 taxa of crops or other cultivated plants, and 83 taxa of allochthonous plants not present in Bolòs et al. [14] were not included. The latter, in a certain sense, are indeed a part of the territorial ethnoflora but were not analyzed in the present study.

Data analysis of the ethnoflora was carried out in two ways due the lack of correspondence between physiographic zones and administrative divisions (districts). Some of the prospected areas were difficult to incorporate into a single physiographic category, since the division by districts is not only based on geographic characteristics, but also on socioeconomic factors.

In the first method, six physiographic zones according to Bolòs et al. [14] were considered in order to analyze in which of them the plants mentioned by our informants grow.

In the second method, the ethnobotanical data from 21 studied territories were grouped into three major areas (Pyrenean, inland, and littoral) (Table 1) to test if there are differences between them.

**Table 1:** Three major geographic areas in Catalonia and ethnobotanical studies carried out in each one

Territory	Studied areas (references)
Pyrenean and pre-Pyrenean territories (abridged Pyrenean)	Alt Urgell [26], Cerdanya [27, 28], Garrotxa [29], Pallars Jussà and Pallars Sobirà [30], Ripollès [31], and Vall d’Aran [32]
Inland territory (inland)	Garrigues and a part of Segrià [33], Sant Feliu Sasserra [34], and Segarra [35]
Littoral and pre-littoral territories (littoral)	Anoia [36], Alt Empordà [37], Baix Llobregat [38], Gallecs [39], Gavarres [40], Gironès [41], Guilleries [42], Montseny [43], Serra de Collserola [44], Serra de Prades [45], Ulldemolins [46], and Vall del Tenes [47]

Some biases may have occurred as a result of the size of the sampled zones and the intensity of prospection, especially when the three main geographical areas are considered.

In addition, assuming that different plant biotypes are preferentially linked to different geographic and climatic areas, another comparative study was composed of plant life forms. The evaluation of the taxa availability throughout the year was done by comparing the ethnobotanical data with Raunkjaer’s main categories, as indicated in Bolòs et al. [14] for each taxon. When one taxon presented more than one life form, the predominating one was adopted. A chi-square test was carried out to compare the life forms in the three major geographic areas. The Fisher’s exact test was used when some categories demonstrated frequencies below 5. Both tests were carried out using XLSTAT software (v.2014.5.3, Addinsoft SARL).

Finally, with the aim of assessing the general state of ethnobotanical knowledge in the studied area, the ethnobotanicity index (EI) [48] was calculated. EI is the quotient between the number of plants used (here taking into account native plants) and the total number of plants that constitute the flora of the territory, expressed as a percentage. For this purpose, only the plants present in the Catalan linguistic area’s flora [14] were considered, and 3475 taxa (as the number of autochthonous taxa in Catalonia [49]) were adopted. The informant consensus factor (F_IC_) [50] was also calculated to evaluate the level of homogeneity, in terms of informants’ agreement in the same uses, among the information. This is the ratio of the number of use reports (hereinafter, UR) minus the number of used taxa to the number of UR minus one. This factor is more reliable when closer to 1.

The statistical analyses, including descriptive statistics, were carried out with Excel (Microsoft Excel 2007). The averages of total plants (TP/I), medicinal plants (MP/I), food plants (FP/I), plants with other uses (OUP/I), in all cases divided by the number of informants, and the linguistic diversity phytonymic index (LDPI) [51] were calculated for the three major areas. STATA 10.1 for Windows (Stata Corporation, TX, USA) was used for the one-way ANOVA and Bonferroni test in order to check the statistical differences among territories. A Shapiro–Wilk test was carried out to check the data normality.

## Results and discussion

### Physiographic distribution of the ethnoflora

**Fig. 2:**
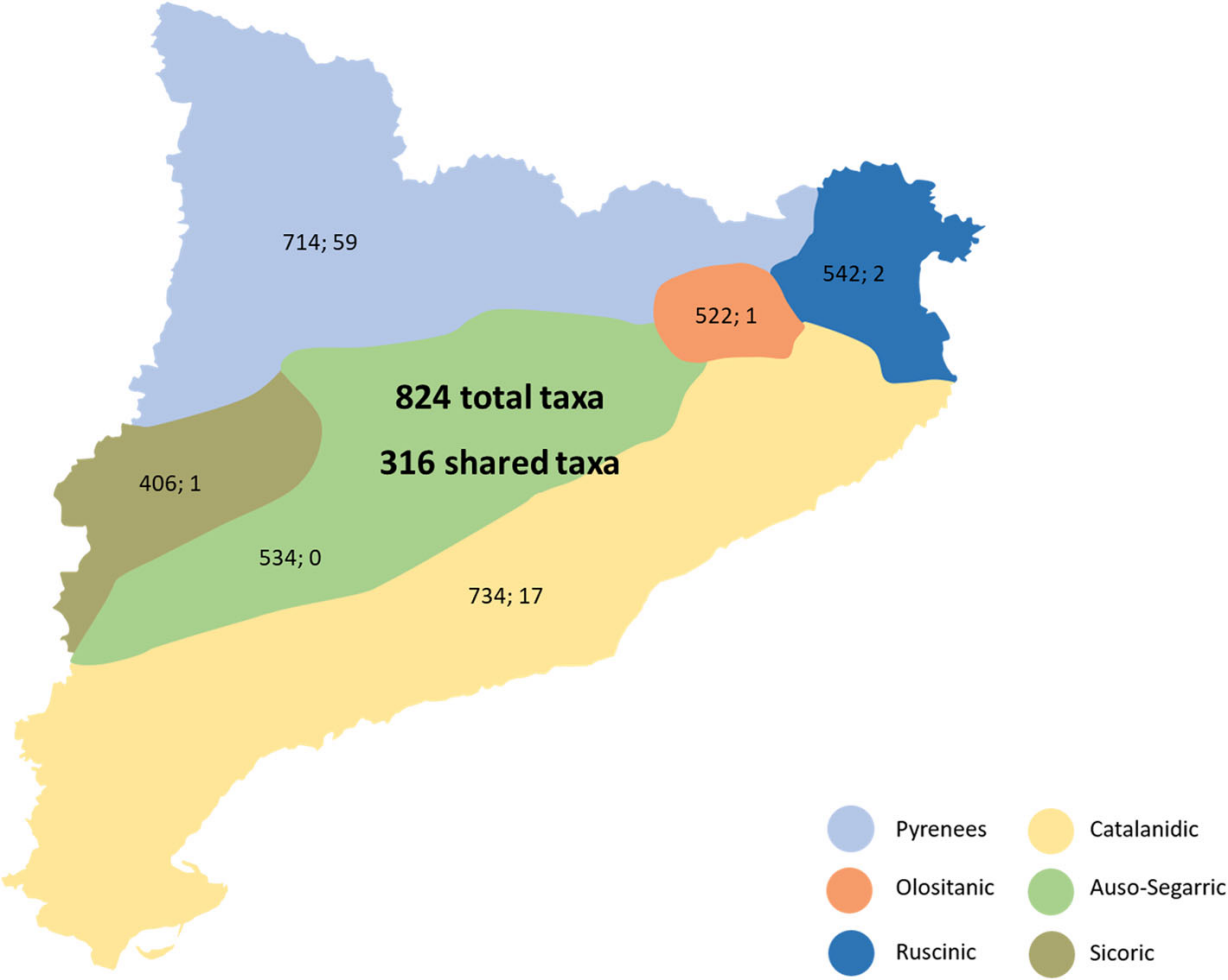
Total and exclusive taxa of the ethnoflora growing in each of the six physiographic zones. In bold, the number of taxa for all Catalonia and the number of taxa shared by the six physiographic zones

In order to evaluate the occurrence of the ethnoflora reported by the informants, the distribution of the 824 taxa in the six physiographic zones was analyzed. A total of 316 taxa, representing 38.35% of the ethnoflora, are shared by all six zones. Therefore, more than one third of the taxa recorded as useful are available to all the informants within the present analysis. This is in agreement with the idea proposed by Johns et al. [52] regarding a relationship between the abundance and availability of a plant resource and its effective use. If we observe the numbers for each territory (Fig. 2; Appendix Table 2), it logically appears that both of the most extensive physiographic zones potentially host a higher number of taxa. In parallel, zonal intersections exist (Appendix Table 2). The number of species shared by two (Pyrenees and Catalanidic) or three (the previous two plus Olositanic) zones is high, since the Catalanidic zone comprises the Prelittoral range, and the Olositanic zone includes the territory of Alta Garrotxa, both sharing plants with the Pyrenees. The species relationship between the Ruscinic and Catalanidic zones, which is also high, is comprehensible due to their both sharing the coastal area, with a similar flora. If the Ruscinic zone is added to the latter two zones, the coincidence in taxa is also logical, since the Ruscinic zone, with mountains of up to 1500 m and littoral plain areas, is a kind of hinge between the Pyrenees and the Catalanidic zone. The degree of coincidence is also high when considering the intersection of four zones, all but the Olositanic and Sicoric zones, and the intersection of five zones, all but the Sicoric zone, the latter with a quite specialized flora conditioned by the arid or semi-arid climate. Surprisingly, no intersection was found between the Auso-Segarric and Sicoric zones, even though they share physiographic and floristic traits.

### Distribution of the ethnoflora in the three major geographic areas

**Fig. 3:**
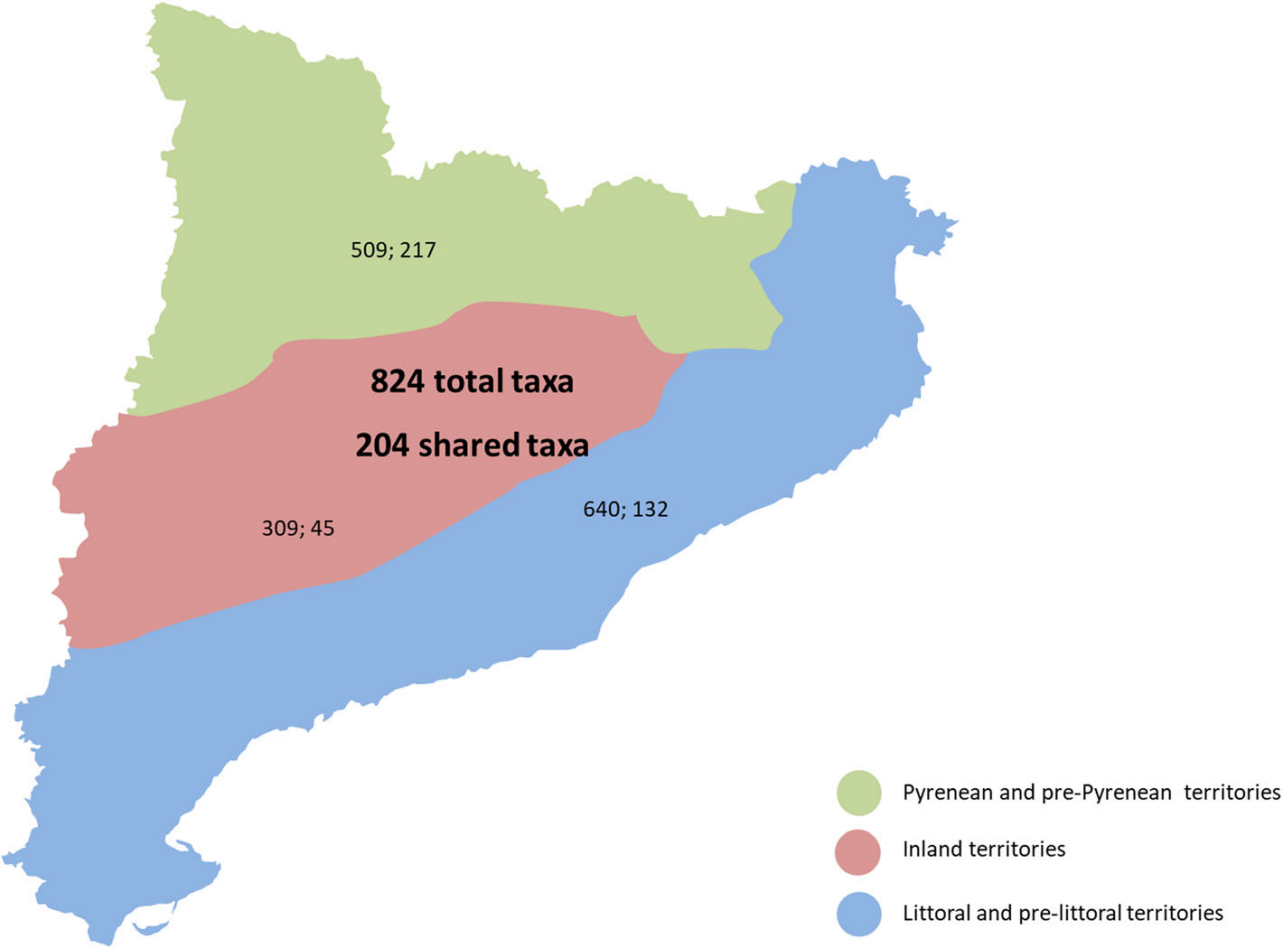
Total and exclusive taxa quoted in each of three major geographic areas. In bold, the number of taxa for all Catalonia and the number of taxa shared by these three areas

Out of the 824 autochthonous taxa currently recorded in the Catalan ethnoflora, 509 were reported in the Pyrenean territory, 309 in the inland territory, and 640 in the littoral territory (Fig. 3). It is not strange that the Pyrenean and littoral territories present the highest number of taxa, since they are the most ethnobotanically prospected areas. The inland territory has been, until very recent times, scarcely prospected [35]. Ethnofloristic studies performed in the arid districts of Garrigues and a part of Segrià [33], and in the district of Bages [34] are beginning to fill the existing gaps.

A total of 394 taxa (47.82%) were reported in only one out of the three areas, of which 217 are exclusive to the ethnoflora reported in the Pyrenean territory, 132 in the inland territory, and 45 in the littoral territory. Taxa were reported as useful in one or another territory, irrespective of whether they grow there or not. The following species can be indicated as being typical examples of each major geographic area: *Achillea ptarmica* L. subsp. *pyrenaica* (Sibth. ex Godr. in Gren. et Godr.) Rouy, *Alkanna tinctoria* Tausch, *Meum athamanticum* Jacq. subsp. *athamanticum*, and *Trifolium alpinum* L., only quoted as inhabiting the Pyrenean territory; *Artemisia herba-alba* Asso, *Plantago albicans* L., *Moricandia arvensis* (L.) DC., and *Salsola vermiculata* L., only in the inland territory; and *Centaurium erythraea* Rafn subsp. *erythraea*, *Crithmum maritimum* L., *Myrtus communis* L., and *Saxifraga vayredana* Luiz., only in the littoral territory.

The 430 remaining taxa (52.18%) correspond to several intersections of taxa reported in more than one territory (Appendix Table 3), 204 of which are shared by all three major areas, and of these, 121 (59.31%) were naturally distributed throughout the territory. Some examples of plants reported in all territories are as follows: *Achillea millefolium* L., *Aconitum napellus* L. susbp. *vulgare* Rouy et Fouc., *Arnica montana* L. subsp. *montana*, *Aphyllanthes monspeliensis* L., *Arbutus unedo* L., *Asparagus acutifolius* L., *Celtis australis* L., *Rosmarinus officinalis* L., *Sambucus nigra* L., and *Thymus vulgaris* L., the two latter being the most cited in ethnobotanical prospections in Catalonia. Of these, *Aconitum napellus* and *Arnica montana* subsp. *montana* grow only in the high mountains, but they are known and referenced in all of the areas.

Concerning the intersections of two territories, the one between the Pyrenean zone and the littoral zone is the highest, with 166 taxa.

Some of these interactions between territories are related with the ancient trade of medicinal plants and other products. This was practiced by women called “*trementinaires*” (from “*trementina*”, turpentine) and served to provide people in lowland areas with some typically Pyrenean material, acting from north to south, from the high mountains to the lowlands [53]. Nowadays, some of these exchanges are still present but in a different form. The most typical example is *Arnica montana* subsp. *montana*. This is only present in the Pyrenean territory as mentioned before, but it is known and claimed as useful in all the prospected areas, with people often collecting this plant in the Pyrenees or purchasing it in herbal shops. Conversely, it is not as common to find species that are exclusive to the lowlands used in the whole territory. This may be, at least in part, because these plants may also be found growing in the low parts of the Pyrenean territory.

### Ethnoflora and life forms

**Fig. 4:**
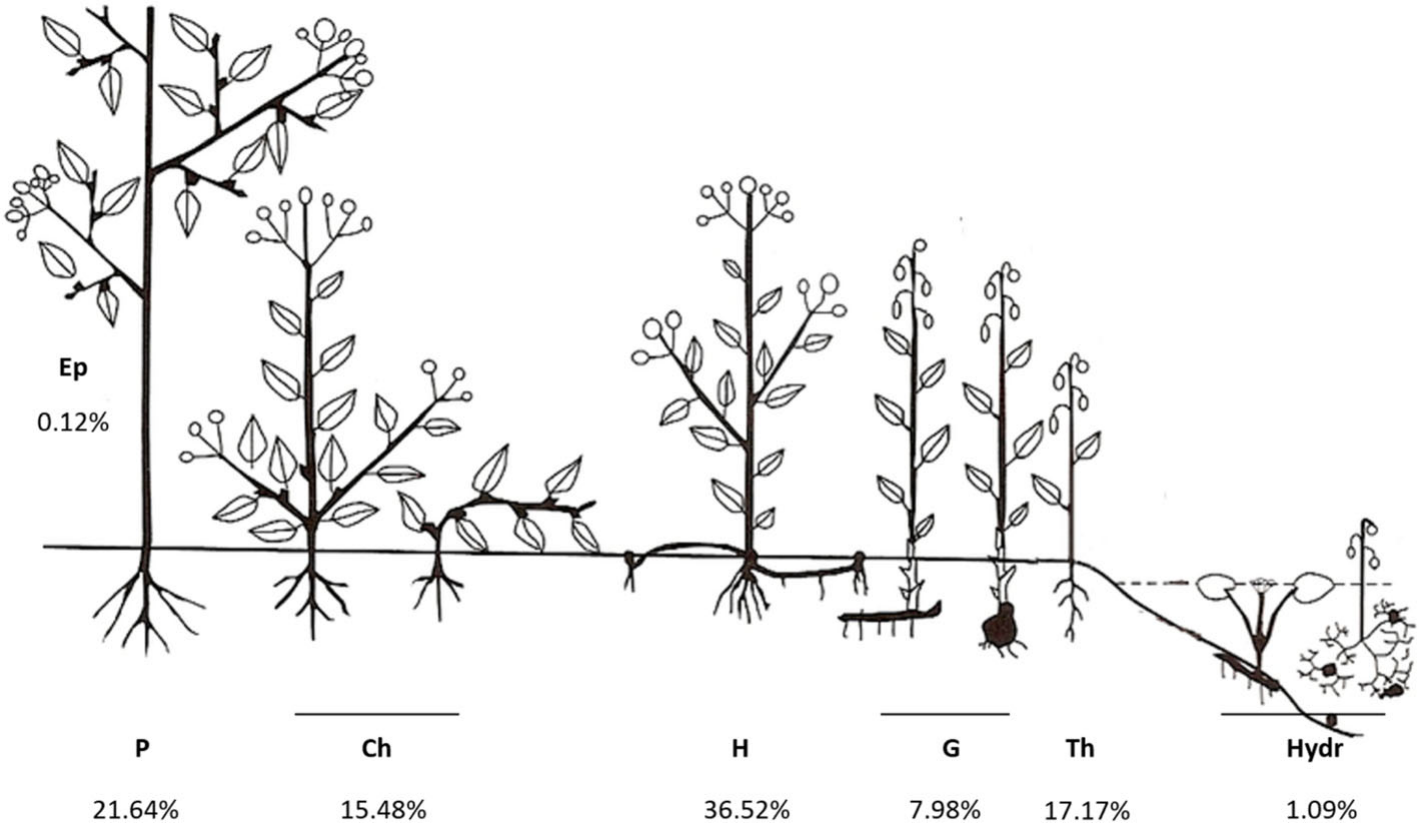
Life form percentages of the ethnoflora of Catalonia. Ep, epiphytes; P, phanerophytes; Ch, chamaephytes; H, hemicryptophytes; G, geophytes; Th, therophytes; Hydr, hydrophytes

In order to analyze the plant life forms in Catalonia’s ethnoflora, the 824 taxa with folk uses were grouped according to Raunkjaer’s classifications [18]. The predominant forms are hemicryptophytes with 36.52%, followed by phanerophytes (21.64%), including macrophanerophytes and nanophanerophytes; therophytes (17.17%); chamaephytes (15.48%); geophytes (7.98%); and in the last positions, i.e., practically not reported as being a useful flora, hydrophytes (1.09%) and epiphytes (0.12%) (Fig. 4).

In agreement with the intuition and the ease of availability theory by Johns et al. [52], phanerophytes and chamaephytes, presents all year round, and hemicryptophytes, only absent in unfavorable conditions, represent the major part of the useful flora. Those taxa with biomass present most of the year through are more predominant than therophytes, annual plants, available only in a determined period of the year. Among the hydrophytes, with only eight taxa, two highly appreciated and reported food plants are found: *Apium nodiflorum* (L.) Lag. and *Rorippa nasturtium-aquaticum* (L.) Hayek. The epiphytes are only represented by *Viscum album* L., a very well-known medicinal plant, but one that was not reported exclusively for this purpose. Thus, the two last categories are really minor in terms of the number of taxa, but contain some frequently reported species.

**Fig. 5:**
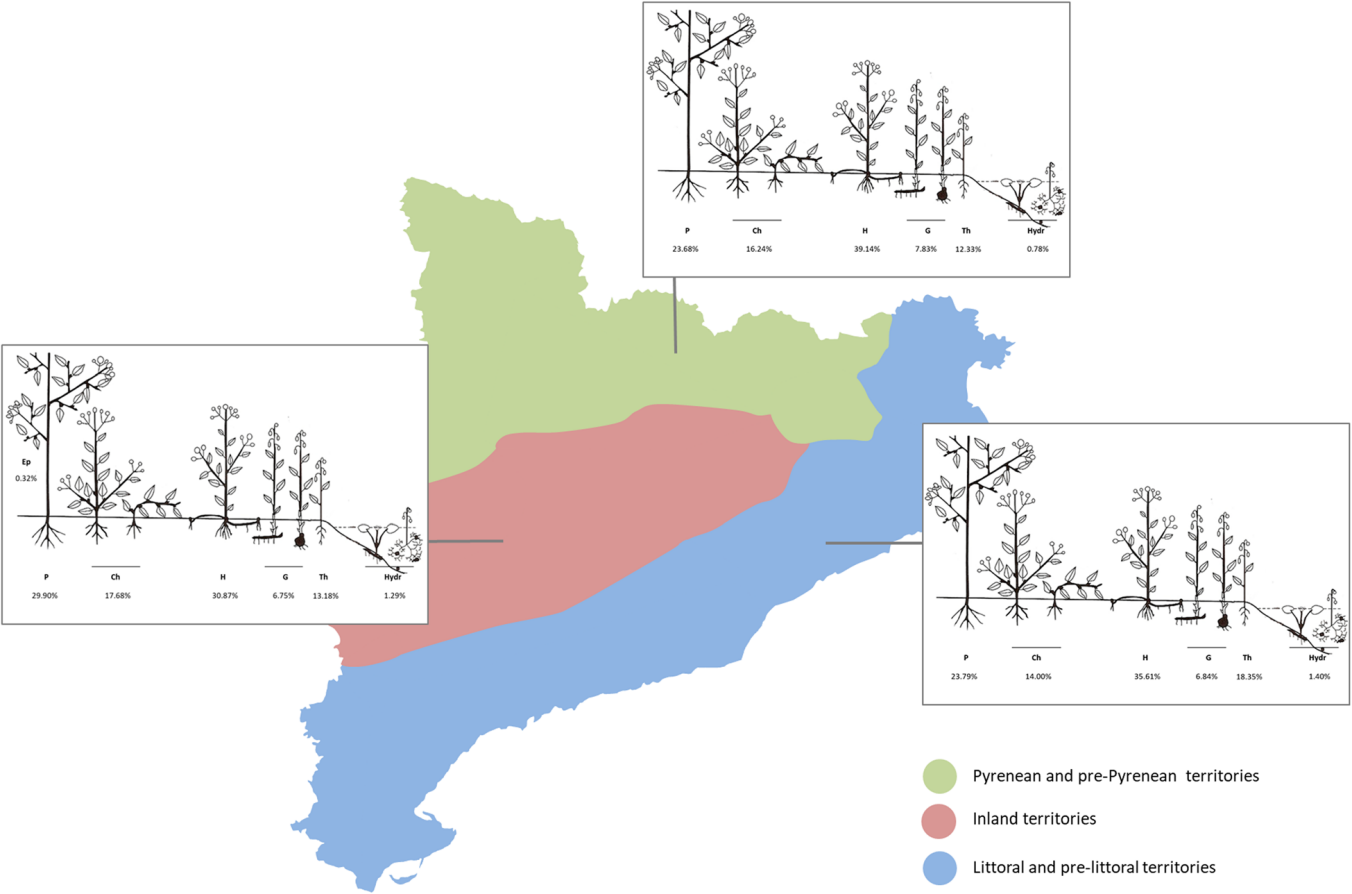
Life form diagrams for each of the three major areas. Ep, epiphyte; P, phanerophyte; Ch, chamaephyte; H, hemicryptophyte; G, geophyte; Th, therophyte; Hydr, hydrophyte

Analyzing the life forms in the three major areas (Fig. 5), the chi-square test shows statistically significant differences among these areas (*χ*^2^ = 22.4885; p = 0.0324). The Fisher’s exact test shows a low observed frequency for hemicryptophytes in the inland territory. Contrarily, the observed frequency of the phanerophytes was higher than what was expected in this territory. This result could be explained by the predominance of the shrubs and subshrubs in the plant landscape of the arid and semi-arid areas. Regarding the therophytes, the observed frequencies were lower than expected in the Pyrenees and higher in the littoral territories. No explanation was found for these results.

### Quantitative ethnobotany

The ethnobotanicity index in Catalonia as a whole is, at present, 23.71%, roughly indicating that between one fifth and one quarter of the plants in the territory have some popular use. The EI value is similar to other well-studied areas in the Iberian Peninsula [54–56]. The informant consensus factor (F_IC_) for all plant uses throughout Catalonia is 0.99, very close to the maximum value of 1.00, suggesting a very high reliability in terms of uses and a robust traditional knowledge.

**Fig. 6:**
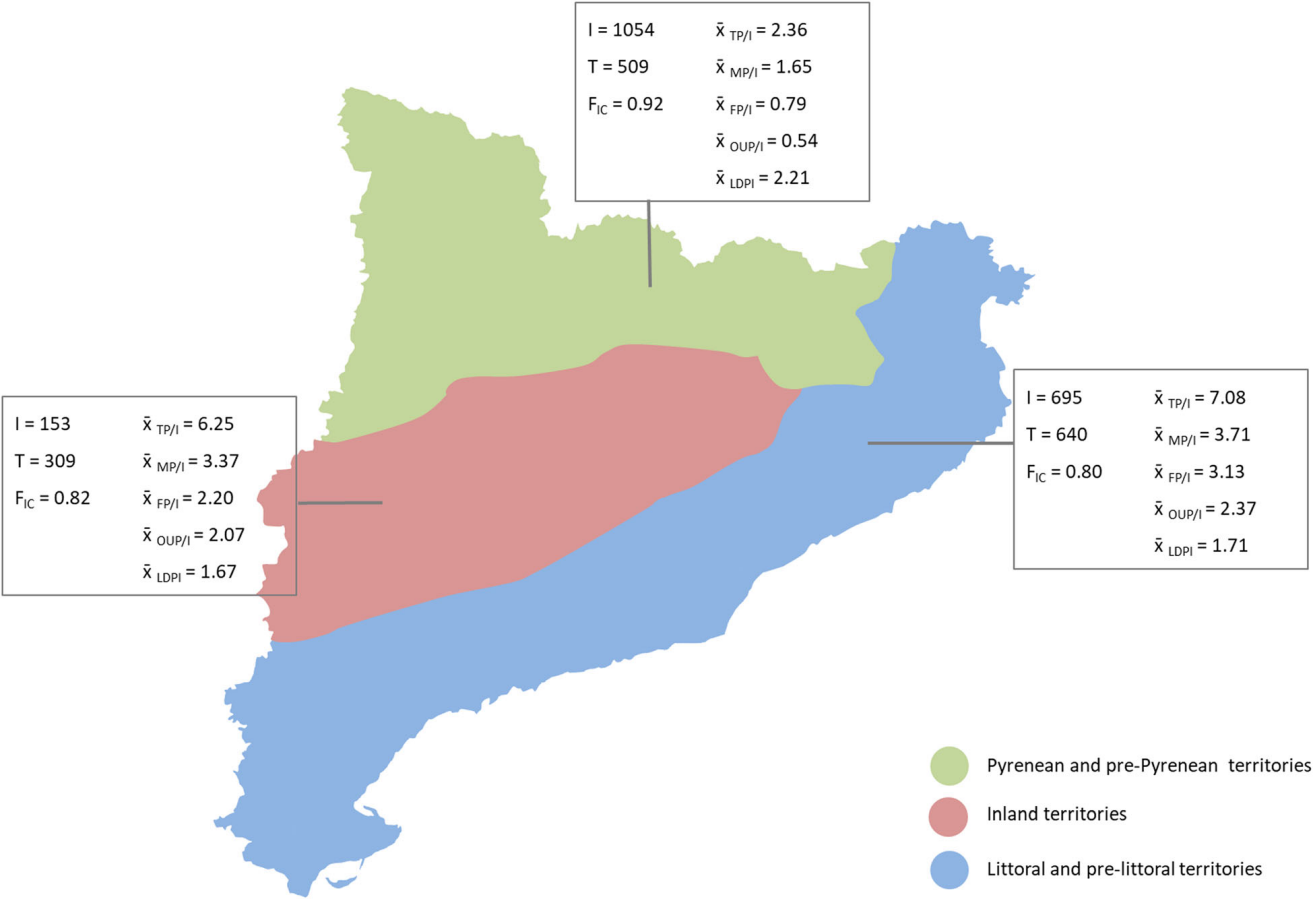
Mean values of ethnobotanical quantitative parameters for each of the three major areas. I, informants; T, taxa; F_IC_, informant consensus factor; TP/I, total plants divided by number of informants; MP/I, medicinal plants divided by number of informants; FP/I, food plants divided by number of informants; OUP/I, plants with other uses divided by number of informants; LDPI, linguistic diversity phytonymic index

Finally, a summary of descriptive statistics for the three major geographic areas is shown in Fig. 6. At first glance, only slight differences among the territories for these parameters were observed. Data are normally distributed according to the Shapiro–Wilk test. Statistically significant differences in total plants per informant (TP/I; *F* = 4.03; *p* = 0.0357), food plants per informant (FP/I; *F* = 4.14; *p* = 0.0332), and plants with other uses per informant (OUP/I; *F* = 4.57; *p* = 0.0248) were revealed by the one-way ANOVA test. Despite these results, significant differences were only found between Pyrenean and littoral territories, the two most intensively sampled areas. This bias may be due to the size of the studies carried out in these areas. The increase in the number of informants and the number of plants follow a non-linear relationship and a saturation effect can usually be detected.

## Conclusions

This study analyzed the Catalan ethnoflora and the life forms of its plants within a physiographic and geographic framework and demonstrated the particular importance of plants with a large distribution area and plants with available biomass throughout the year. Nevertheless, plants that exclusively grow in one area, or plants only temporarily present, are also relevant in the ethnoflora and may be of particular local interest.

The ethnobotanicity index shows that between one fifth and one quarter of the flora of Catalonia is traditionally reported and used. The informant consensus factor (F_IC_) obtained indicates a high, homogeneous level of ethnobotanical information, verifying the reliability of the data recorded. This study constitutes a new approach for the analysis of the ethnobotanical data. Now applied to data collected in Catalonia, it could be useful, for further comparisons, to see the results of a similar analysis in other geographic areas.

### Supplementary Information

**Additional file 1.** Supplementary material.

## Data Availability

All data are available in the corresponding author email airy.gras@ibb.csic.es.

## Appendix

**Table 2:** Number of taxa shared by physiographic zones. 1, Pyrenees; 2, Ruscinic; 3, Olositanic; 4, Catalanidic; 5, Auso-Segarric; 6, Sicoric

Physiographic zones intersections	Number of taxa
1∩2	4
1∩3	8
1∩4	39
1∩5	4
2∩4	32
4∩5	4
4∩6	4
1∩2∩4	31
1∩2∩5	2
1∩2∩6	1
1∩3∩4	48
1∩3∩5	1
1∩4∩5	15
1∩4∩6	2
2∩3∩4	4
2∩4∩5	6
2∩4∩6	18
1∩2∩3∩4	13
1∩2∩3∩5	2
1∩2∩4∩5	15
1∩2∩4∩6	5
1∩3∩4∩5	52
1∩3∩4∩6	3
1∩4∩5∩6	7
2∩3∩4∩5	4
2∩4∩5∩6	11
1∩2∩3∩4∩5	54
1∩2∩3∩4∩6	2
1∩2∩4∩5∩6	20
1∩3∩4∩5∩6	5
2∩3∩4∩5∩6	9
1∩2∩3∩4∩5∩6	316

**Table 3:** Intersection of taxa among major territories. 1, Pyrenean territory; 2, inland territory; 3, littoral territory

Territories intersection	Number of taxa
1∩2	11
1∩3	166
2∩3	49
1∩2∩3	204
